# Antimicrobial photodynamic therapy for severe acute radiodermatitis in patients undergoing head and neck radiotherapy: a case series

**DOI:** 10.3389/fonc.2025.1746492

**Published:** 2026-01-21

**Authors:** Isabella Macedo Costa e Silva, Carolina de Souza Custódio, Mylene Martins Monteiro, Karina Alexandra Batista da Silva Freitas, Talita Oliveira de Lima, Wenzel Castro de Abreu, Ricardo Gomes dos Reis, Eliete Neves Silva Guerra, Elaine Barros Ferreira, Paula Elaine Diniz dos Reis

**Affiliations:** 1 University of Brasília, School of Health Sciences, Interdisciplinary Laboratory of Research Applied to Clinical Practice in Oncology (LIONCO), Brasília, Brazil; 2 University Hospital of Brasília, Brasília, Brazil; 3 University of Brasília, School of Health Sciences, Laboratory of Oral Histopathology, Brasília, Brazil; 4 Hospital das Clínicas of the Botucatu Medical School at the São Paulo State University, São Paulo, Brazil

**Keywords:** acute radiodermatitis, antimicrobial photodynamic therapy, case series, head & neck, radiotherapy

## Abstract

**Introduction:**

Acute radiodermatitis (ARD) is a frequent and debilitating inflammatory toxicity in patients undergoing head and neck radiotherapy (RT). In severe cases, ARD may progress to moist desquamation, with the potential for ulceration. Colonization or infection with *Staphylococcus aureus* is a critical independent risk factor for worsening ARD. Antimicrobial photodynamic therapy (aPDT) has emerged as a promising adjunctive therapeutic approach, with potential to reduce microbial burden, modulate inflammation, and accelerate tissue repair. This study aimed to evaluate the clinical effects of aPDT in patients with severe ARD.

**Methods:**

We conducted a case series, according to the CARE Statement Guideline, over an eight-month period involving five patients with severe ARD (GRA-L scale ≥ 5) who received aPDT following a standardized protocol. The protocol involved the topical application of methylene blue 1% as the photosensitizer followed by irradiation with a 660-nm diode laser delivering an energy density of 91.836 J/cm^2^ per point, with sessions repeated every 72 hours. Clinical assessments performed at the same interval and included photographic documentation and severity scoring with the GRA-L scale.

**Results:**

aPDT led to significant clinical improvement in all five cases. ARD severity decreased from a mean initial grade of 6 (range: 5–8) to grade 1 in all patients, with no progression to more advanced grades after therapy initiation. Clinical stability was maintained until completion of the prescribed RT course, including the case in which aPDT was initiated during treatment. In the post-RT cases, recovery occurred within 6 to 15 days after aPDT initiation. No adverse effects related to aPDT were observed.

**Conclusions:**

This case series provides preliminary evidence that aPDT may assist in the management of severe ARD in patients undergoing head and neck RT. While the observed improvements were encouraging, these findings are exploratory and require confirmation in larger, controlled studies before aPDT can be considered within standardized supportive care approaches.

## 1 Introduction

Radiotherapy (RT) is a cornerstone in the management of cancers that affect the head and neck region, typically using ionizing radiation doses ranging from 60 Grays (Gy) to 70 Gy (1). Despite its efficacy, RT is associated with a significant incidence of acute toxicities that can substantially impair patients’ quality of life (2). In this context, acute radiodermatitis (ARD) is a common cutaneous adverse event characterized by an inflammatory response to ionizing radiation (3–5).

Early manifestations of ARD include transient erythema within the first 24 hours after RT initiation, followed by hyperpigmentation, pain, itching, edema, and desquamation (6, 7). In severe cases, ARD may progress to moist desquamation, ulceration, or even necrosis (6, 8, 9).

Pathophysiologically, ARD results from radiation-induced depletion of basal keratinocytes, which compromises epidermal regeneration (5, 10). Repeated radiation fractions exacerbate these effects, leading to increased transepidermal water loss, disruption of the cutaneous microbiome, and a greater susceptibility to colonization by opportunistic pathogens, particularly in areas affected by moist desquamation (9, 11–14).

A critical independent risk factor for the development of severe ARD is colonization and infection by *Staphylococcus aureus* (12). Furthermore, depletion of commensal bacteria such as *S. hominis* and *S. epidermidis* often precedes this dysbiosis (12). This observation is complemented by recent microbiome evidence demonstrating that RT, particularly hypofractionated regimens, induces dysbiosis by increasing the abundance of potentially pathogenic genera, including *Finegoldia* and *Dermacoccus* (15). At the same time, a relative increase in *Staphylococcus* is associated with worsening clinician-reported pruritus and dermatitis (15). Given that head and neck RT protocols are particularly aggressive, patients may be more vulnerable to these opportunistic infections. Thus, targeting *S. aureus* and restoring microbial homeostasis represent a key therapeutic strategy.

Despite the clinical burden of ARD, there is currently no standardized management protocol, largely due to heterogeneity across studies and interventions, including topical agents, corticosteroids, barrier dressings, and natural therapies such as chamomile (3, 4, 16). In this context, antimicrobial photodynamic therapy (aPDT) has emerged as a promising adjunct approach (17–19). By activating photosensitizers with visible light (from lasers or LEDs) to generate reactive oxygen species (ROS), aPDT induces microbial cell death through oxidative stress (17, 20). This therapy has demonstrated efficacy against a broad spectrum of pathogens, including resistant *S. aureus* strains frequently associated with severe cases of ARD (12).

Among the available photosensitizers, methylene blue (MB) is a cationic phenothiazine dye widely used in aPDT due to its strong absorption in the red-light spectrum (630–680 nm), which favors effective tissue penetration, and its photochemical properties (21, 22). The clinical advantages of MB include its excellent safety profile, widespread availability, relatively low cost, and practical application features such as a short incubation period and generally painless irradiation, making it a suitable agent for managing superficial infections in a clinical setting (17, 22).

Given the multifactorial nature of severe ARD, which involves skin barrier dysfunction, inflammation, and secondary infection, aPDT offers a multimodal mechanism to support local healing and tissue repair. This study reports a case series describing the clinical effects of aPDT in patients with severe ARD undergoing head and neck RT.

## 2 Case descriptions

We conducted a case series over an eight-month period from 2024 to 2025 involving five patients with severe ARD (GRA-L scale ≥ 5) who received aPDT following a standardized protocol. Clinical assessments were performed every 72 hours, including photographic documentation and severity scoring with the GRA-L scale. This study complies with the Declaration of Helsinki, and it has already been approved by the Research Ethics Committee of the University Hospital of Brasília, Brazil. The case description was conducted according to the CARE Statement Guideline (23).

The sociodemographic and clinical characteristics of the five patients are summarized in **Table 1**. All patients developed severe ARD (GRA-L grade ≥ 5) during or after RT and were subsequently treated with aPDT.

**Table 1:** Sociodemographic and clinical characteristics of patients included in the case series (n = 5).

Case	Age	Sex (F/M)	Cancer diagnosis	TNM	Staging	RT	Fractions	TD (Gy)	DD (Gy)	aPDT (n)
1	83	M	Laryngeal SCC	T1N0M0	I	3D-CRT	29	65.25	2.25	3
2	64	M	Hypopharyngeal SCC	T4N2M0	III	3D-CRT	35	70	2	2
3	75	F	Scalp SCC in situ	T2N0M0	II	2D-RT	10	44	4.4	2
4	81	M	Skin retroauricular SCC	TxNxM0	III	3D-CRT	33	66	2	4
5	87	M	Skin thoracic and cervical regions SCC	T4NxMx	IV	3D-CRT	30	60	2	1

3D-CRT: Three-Dimensional Conformal Radiation Therapy; 2D-RT: Two-Dimensional Radiation Therapy; DD: Daily Dose; RT: Radiotherapy; SCC: Squamous Cell Carcinoma; TD: Total Dose. Note: Patient 3 received a hypofractionated regimen 44 Gy in 10 fractions; 4.4 Gy/day, which is a standard schedule for epithelial skin cancers, particularly in elderly patients, supported by evidence demonstrating comparable local control to conventional fractionation while reducing treatment duration (24). TNM staging criteria are based on the American Joint Committee on Cancer (AJCC) Staging Manual, 8th Edition (25).

**Figure 1:**
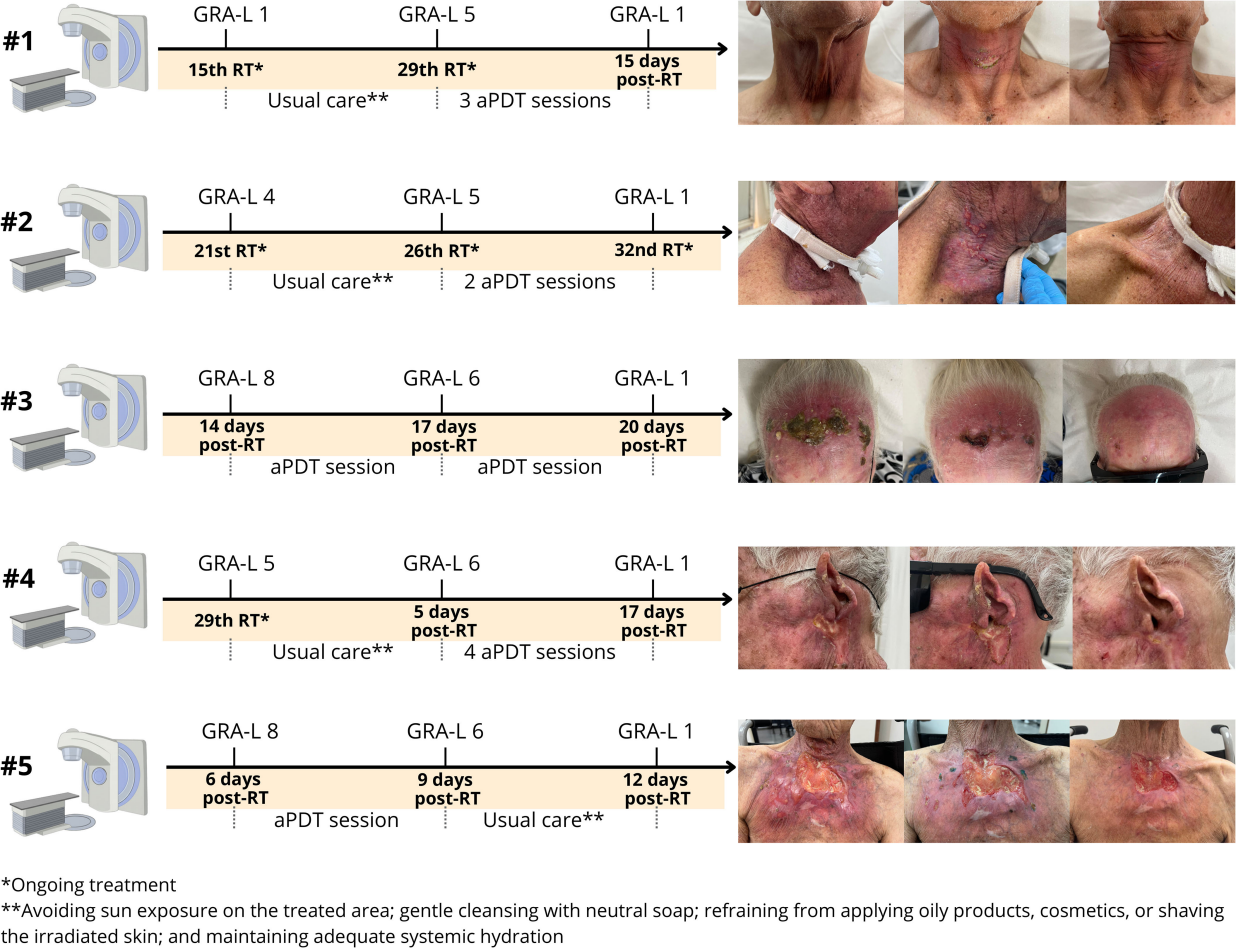
Timeline of the clinical course for five patients with severe ARD treated with aPDT.

The clinical progression of the severe ARD lesions, the appearance during aPDT intervention, and the final healing outcomes for all five patients are presented in **Figure 1**.

### 2.1 Case 1

An 83-year-old man with laryngeal squamous cell carcinoma (SCC) and a history of hypertension and smoking. At the 15th RT fraction (cumulative dose: 33.75 Gy), he developed grade 1 ARD, characterized by discrete erythema in the anterior cervical region, initially managed with institutional usual care. The condition progressed to a grade 5 ARD, with intense erythema and moist desquamation, during the 29th fraction (65.25 Gy), which was the final dose of RT. A total of three sessions of aPDT were administered post-RT at standardized 72-hour intervals. During the first session, the patient reported a burning sensation, which subsided after repositioning of the diode laser device. Complete re-epithelialization and clinical resolution of moist desquamation, achieving a final grade 1 ARD, were confirmed on day 15 post-RT.

### 2.2 Case 2

A 64-year-old man with hypopharyngeal SCC and a history of smoking and alcohol consumption. At fraction 21 (42 Gy), he presented with grade 4 ARD showing erythema and bilateral dry desquamation, for which usual care was administered. By fraction 26 (52 Gy), the condition had progressed to grade 5 ARD with moist desquamation. At this point, aPDT was initiated during the ongoing RT course, and two sessions were performed. This approach led to complete resolution of moist desquamation and improvement to grade 1 ARD by fraction 32 (64 Gy). Subsequently, the RT course was completed without interruption, with no further worsening of skin toxicity observed.

### 2.3 Case 3

A 75-year-old female patient with SCC *in situ* of the scalp and comorbidities including pre-diabetes, hypertension, and extensive solar elastosis. She completed 10 RT fractions (44 Gy), which constituted the full prescribed dose. Fourteen days after RT completion, she presented with grade 8 ARD, characterized by necrosis and a fetid odor. A single aPDT session was initiated immediately, which produced marked improvement within 72 hours, with substantial removal of necrotic tissue and resolution of the odor. Following a second aPDT session, complete wound healing was achieved within one week.

### 2.4 Case 4

An 81-year-old male patient with retroauricular skin SCC. At fraction 29 (58 Gy), he developed grade 5 ARD with moist desquamation, which was managed with institutional usual care throughout the remainder of the RT course. Despite these measures, the condition persisted and progressed to grade 6 ARD five days after RT completion. At that point, aPDT was initiated, and four sessions were performed at 72-hour intervals. Progressive improvement was observed after each session, culminating in substantial clinical improvement.

### 2.5 Case 5

An 87-year-old man with invasive cutaneous carcinoma showing basaloid and squamous differentiation. Six days after completing all 30 RT fractions (total dose: 60 Gy), he presented with grade 6 ARD, characterized by disseminated moist desquamation and bullae formation on the thoracic and cervical regions. aPDT was initiated at this time, and a single first session led to regression of bullae and marked improvement of moist desquamation within 72 hours, resulting in a reduction to grade 6 ARD. Following a second session, complete healing and restoration of normal skin texture recovery were noted.

## 3 Diagnostic assessment and therapeutic intervention

### 3.1 Diagnostic assessment

All patients underwent a standardized clinical examination protocol upon referral. Assessments were performed by a trained examiner through visual inspection and photographic documentation of the irradiated area to monitor the evolution of skin changes. Lesions were graded according to Acute Radiodermatitis Grading (GRA-L) scale, developed by LIONCO/UnB research group: 0 (no change), 1 (erythema/hyperpigmentation), 2 (dry skin), 3 (localized dry desquamation in one or more separate spots), 4 (dry desquamation in one or more contiguous spots), 5 (localized moist desquamation and/or in folds), 6 (disseminated moist desquamation), 7 (bleeding and/or ulceration), 8 (necrosis) (26).

In accordance with the institution’s usual care protocol, all patients received standardized supportive care based on a validated educational manual for head and neck cancer patients undergoing RT (27). This protocol was delivered through nursing consultations and provided structured guidance on proper skin care. Key recommendations included: avoiding sun exposure on the treated area; gentle cleansing with neutral soap; refraining from applying oily products, cosmetics, or shaving the irradiated skin; and maintaining adequate systemic hydration. It is important to note that this standardized protocol did not include the routine use of topical corticosteroids or advanced wound dressings for all patients.

### 3.2 Therapeutic intervention

Following the diagnosis of severe ARD, treatment was initiated according to a standardized aPDT protocol (**Table 2**). Lesions were cleansed with 0.9% saline solution, followed by topical application of methylene blue 1% (pharmacy-compounded formulation) as photosensitizer. After a 5-minute pre-irradiation period, excess dye was removed with sterile gauze.

**Table 2:** Parameters for aPDT protocol.

Category	Specification	Detail
Equipment Information	Equipment	Therapy EC
	Manufacturer	DMC^®^, São Carlos, Brazil
	Emitter Type	Laser diode
	Beam Delivery System	Optical Fiber (InGaA1P)
Irradiation Parameters	Wavelength (nm)	660
	Energy per point (J)	9
	Power (mW)	100
	Spot size area at 1 cm (cm²)	0.098
	Number of emitters	1
	Time (s)	90
	Energy Density (J/cm²)	91.836
	Irradiance (W/cm²)	1.02
	Photon fluence (p.J/cm²)	174.42
	Operation Mode	Continuous

InGaAIP, Indium Gallium Aluminum Phosphide; J, Joule; J/cm², Joules per square centimeter; mW, Milliwatts; W/cm², Watts per square centimeter; E, Einstein.

The aPDT was performed using a diode laser device (Laser Therapy EC, DMC™, São Carlos, Brazil) emitting at 660 nm, which effectively activates methylene blue. The laser beam was applied perpendicularly (90°) to the affected area, covering lesions with adjacent non-overlapping points to ensure homogeneous irradiation. Disposable PVC film protected the laser tip, replaced after each use, and all patients and staff wore specific protective eyewear. Sessions were conducted at 72-hour intervals. The technical irradiation parameters are presented in **Table 2**.

## 4 Discussion

This case series highlights the potential of aPDT as an effective and safe adjuvant therapy for managing severe ARD in cancer patients undergoing head and neck RT. Despite presenting advanced lesions (GRA-L ≥ 5), including necrosis, all patients showed rapid clinical improvement and complete healing of moist desquamation with minimal adverse effects. In this study, “complete healing” referred to full re-epithelialization and clinical resolution of moist desquamation. Mild residual erythema persisted in some patients, corresponding to GRA-L 1 rather than GRA-L 0. These outcomes are clinically relevant, given that severe ARD affects approximately 25% of this population and often leads to interruptions in curative-intent RT (26).

The head and neck region are highly susceptible to severe ARD due to anatomical factors, such as skin folds and the use of aggressive RT protocols (12, 28, 29). Disruption of the skin barrier facilitates microbial colonization, especially by *S. aureus*, which exacerbates ARD severity (12, 13). In addition, patient-related risk factors, including smoking history and actinic elastosis, may impair microvascular function, reduce skin regenerative capacity, and intensify inflammatory responses, thereby increasing vulnerability to more radiation-induced toxicity (30–33). Within this multifactorial context, the dual therapeutic action of aPDT offers a distinct advantage by simultaneously reducing microbial burden and promoting tissue repair (34–36).

aPDT exerts a direct antimicrobial effect and a pro-healing photobiomodulatory effect. Upon activation of methylene blue by red light, ROS are generated and induce oxidative damage to microbial membranes and essential biomolecules, leading to rapid microbial inactivation, an effect consistently demonstrated in *in vitro* and *in vivo* aPDT studies (34, 35). In parallel, sublethal ROS modulate local inflammation and stimulate angiogenesis, fibroblast proliferation, extracellular matrix deposition, and re-epithelialization (36), thereby promoting tissue repair (37). These biological effects are aligned with previous evidence demonstrating aPDT-mediated modulation of matrix remodeling and restoration of tissue integrity (37, 38).

The energy density applied in our protocol (91.836 J/cm²) is consistent with the therapeutic range reported in aPDT studies targeting infected tissues (22, 35, 38). We recognize that clinical trials in superficial infections typically utilize lower energy densities (6–18 J/cm²) in combination with very low MB concentrations (0.0003–0.06 molar) (22). However, higher dosimetric values have proven effective and necessary in deeper or heavily colonized wounds (35, 38). For instance, aPDT delivered at 60 J/cm² using 0.01% MB resulted in clinical improvement of infected diabetic foot ulcers (38), while preclinical murine models of *S. aureus* skin infection demonstrated significant bacterial reduction and accelerated healing at 74 J/cm² (35). Importantly, our protocol employed a substantially higher MB concentration (1%), and higher photosensitizer loads require proportionally greater radiant exposure to ensure adequate photoactivation across the full thickness of inflamed, exudative, and partially necrotic tissue, features that characterize severe ARD.

Importantly, the mechanistic rationale described above was directly reflected in the clinical evolution observed in our case series. The rapid reduction of purulent exudate, foul odor, and necrotic tissue after the first aPDT session mirrors the established antimicrobial and antibiofilm effects of MB (22, 34, 35). Likewise, the marked improvement in erythema, pain, and moist desquamation within 48–72 hours is consistent with the anti-inflammatory and pro-repair roles of sublethal ROS, including modulation of angiogenesis, fibroblast activation, and extracellular matrix remodeling (35, 37).

Beyond clinical healing, the shortened repair time contributed to improved patient satisfaction and quality of life. Severe ARD not only compromises RT delivery but also impacts psychosocial well-being due to body image changes resulting from skin toxicity (39). By accelerating tissue recovery and reducing radiotoxicity, aPDT may serve as a valuable adjunct to standard supportive care in this vulnerable population.

Nevertheless, certain limitations must be acknowledged. Microbiological confirmation of infection was not performed, and the diagnosis relied instead on clinical signs such as foul odor and necrotic appearance. The sample size limits the extrapolation of the findings to broader populations, and the case series design, lacking a control group, precludes robust causal inferences. Future controlled trials with larger cohorts are necessary to validate these promising results and establish evidence-based protocols.

The primary “take-away” lessons from this case series are that long-term effects of head and neck RT contribute significantly to the overall cost of survivorship care. Thus, aPDT can substantially mitigate the clinical and cost-of-care impact for patients, also it would reduce costs associated with utilization of health care resources.

## 5 Conclusion

This case series provides preliminary evidence that aPDT may assist in the management of severe ARD in patients undergoing head and neck RT. While the observed improvements were encouraging, these findings are exploratory and require confirmation in larger, controlled studies before aPDT can be considered within standardized supportive care approaches.

## Data Availability

The original contributions presented in the study are included in the article/supplementary material. Further inquiries can be directed to the corresponding author.
